# Adaptation of the brief psychoeducational intervention programme (BREF) for carers of patients with eating disorders

**DOI:** 10.1192/j.eurpsy.2024.408

**Published:** 2024-08-27

**Authors:** E. Scanferla, J. de Salle, P. Gorwood

**Affiliations:** ^1^GHU Paris psychiatrie et neurosciences, Paris, France

## Abstract

**Introduction:**

Even though international guidelines suggest that psychoeducation for carers should be provided systematically, it remains insufficiently available in psychiatry (1), including for eating disorders (EDs). The complicated interplay of factors contributing to the maintenance of EDs, including family/carer influences, highlights the importance of carer interventions within ED treatment (2). Carer interventions demonstrate positive outcomes for carers themselves, though are also hypothesised to benefit the patient indirectly. The BREF programme is a short, early and systematic single-family psycho-educational programme. The BREF programme is already proven to be effective for other mental disorders (3)

**Objectives:**

The aim of this study is to adapt the BREF programme to the specific needs of carers of patients eating disorders (ED).The main objective was to identify the issues in the experience of the disorder that are most important to carers and which should be the focus of the BREF programme for cares of patients with eating disorders.

**Methods:**

Twenty-eight topics relating to difficulties commonly encountered by carers of patients with eating disorders were identified by a group including mental health professionals with expertise in these disorders, patients and their relatives. The topics tested are illustrated by 2 decks of cards presented to the participants; the first concerns the problems frequently encountered by users living with ED and the other the problems frequently encountered by their carers. The 2 decks were tested by the participants to the first 15 sessions of the BREF ED programme run from January to July 2023 as part of the pilot conducted in a university-hospital department specialised in eating disorders.

**Results:**

30 participants participanted in the study.

The 10 most frequently selected topics relating to patient problems were, in descending order: relative’s fear and anxiety; relative’s false-self functioning; ambivalence towards care; dysmorphophobia; food restrictions; relative’s hyperactivity; eating disorders; denial of symptoms; perfectionism; malnutrition. The 10 most frequently selected topics concerning the issues of carers were: social withdrawal; difficulty navigating care; fatigue/helplessness; disruption to family life as a result of the illness; guilt; right attitude to have with the ill relative; fear for your loved one’s future; fragility of the relative; not knowing/understanding the care their relative is receiving; cost of care and food expenses.

**Conclusions:**

The priority topics highlighted in this study helped to identify relevant content for the BREF programme adapted to the context of eating disorders. This programme appears to be a promising way of responding to the concerns and information needs of carers of patients with ED. In this regard, it addresses a major shortcoming in the organisation of mental health services.

**Disclosure of Interest:**

None Declared

